# The response to fasting and refeeding reveals functional regulation of lipoprotein lipase proteoforms

**DOI:** 10.3389/fphys.2023.1271149

**Published:** 2023-10-16

**Authors:** Pere Carulla, Míriam Badia-Villanueva, Sergi Civit, Montserrat Carrascal, Joaquin Abian, David Ricart-Jané, Miquel Llobera, Albert Casanovas, M. Dolores López-Tejero

**Affiliations:** ^1^ Departament de Bioquímica i Biomedicina Molecular, Facultat de Biologia, Universitat de Barcelona (UB), Barcelona, Spain; ^2^ Departament de Genètica, Microbiologia i Estadística, Facultat de Biologia, Universitat de Barcelona (UB), Barcelona, Spain; ^3^ Biological and Environmental Proteomics, Institute of Biomedical Research of Barcelona, Spanish National Research Council, Institut d’Investigacions Biomèdiques August Pi i Sunyer (IIBB-CSIC/IDIBAPS), Barcelona, Spain

**Keywords:** lipid metabolism, lipoprotein lipase, proteoform, tissue-specific regulation, fasting, white adipose tissue, 2D electrophoresis

## Abstract

Lipoprotein lipase (LPL) is responsible for the intravascular catabolism of triglyceride-rich lipoproteins and plays a central role in whole-body energy balance and lipid homeostasis. As such, LPL is subject to tissue-specific regulation in different physiological conditions, but the mechanisms of this regulation remain incompletely characterized. Previous work revealed that LPL comprises a set of proteoforms with different isoelectric points, but their regulation and functional significance have not been studied thus far. Here we studied the distribution of LPL proteoforms in different rat tissues and their regulation under physiological conditions. First, analysis by two-dimensional electrophoresis and Western blot showed different patterns of LPL proteoforms (i.e., different pI or relative abundance of LPL proteoforms) in different rat tissues under basal conditions, which could be related to the tissue-specific regulation of the enzyme. Next, the comparison of LPL proteoforms from heart and brown adipose tissue between adults and 15-day-old rat pups, two conditions with minimal regulation of LPL in these tissues, yielded virtually the same tissue-specific patterns of LPL proteoforms. In contrast, the pronounced downregulation of LPL activity observed in white adipose tissue during fasting is accompanied by a prominent reconfiguration of the LPL proteoform pattern. Furthermore, refeeding reverts this downregulation of LPL activity and restores the pattern of LPL proteoforms in this tissue. Importantly, this reversible proteoform-specific regulation during fasting and refeeding indicates that LPL proteoforms are functionally diverse. Further investigation of potential differences in the functional properties of LPL proteoforms showed that all proteoforms exhibit lipolytic activity and have similar heparin-binding affinity, although other functional aspects remain to be investigated. Overall, this study demonstrates the ubiquity, differential distribution and specific regulation of LPL proteoforms in rat tissues and underscores the need to consider the existence of LPL proteoforms for a complete understanding of LPL regulation under physiological conditions.

## Introduction

Lipoprotein lipase (LPL EC.3.1.1.34) is a glycoprotein enzyme present in almost all tissues except the liver. The enzyme is synthesised by parenchymal cells, transported across the capillary endothelium by glycosylphosphatidylinositol-anchored high density lipoprotein binding protein 1 (GPIHBP1) and located in its active form on the luminal surface of endothelial cells. In this location, it binds to GPIHBP1 and heparan sulphate proteoglycans (HSPG) through hydrophobic and electrostatic interactions (He et al., 2018; Birrane et al., 2019). The main function of LPL is the hydrolysis of circulating triglycerides (TAG) from chylomicrons and very low density lipoproteins (VLDL) into fatty acids and 2-monoacylglycerol. These fatty acids are taken up by underlying cells and stored in the form of TAG as a nutrient reservoir in white adipose tissue (WAT), catabolized for energy production in heart and skeletal muscle or used as a substrate for thermogenesis in brown adipose tissue (BAT) (Mead et al., 2002). To channel fatty acids towards specific tissues, LPL is subject to tissue-specific regulation in different physiological conditions. For instance, LPL activity in WAT and skeletal muscle are inversely regulated during exercise (Seip et al., 1995), stress (Ricart-Jané et al., 2005), cold exposure (Begin Heick and Heick, 1977; Jensen et al., 2008), and fasting (Ruge et al., 2005). More specifically, in response to fasting, LPL activity is markedly decreased in WAT and concomitantly increased in heart and skeletal muscle (Quig et al., 1983; Doolittle et al., 1990; Bergö et al., 1996a). Furthermore, LPL is highly upregulated in adipocytes after breaking fasting (Eckel et al., 1984; Semenkovich et al., 1989), showing the reversibility of the fasting-mediated downregulation of LPL activity in WAT.

The mechanisms that enable tissue-specific regulation of LPL activity span from the control of LPL gene expression to the release of the enzyme from its functional location in the capillary endothelium (Preiss-Landl et al., 2002). Indeed, in addition to transcriptional and post-transcriptional regulation, LPL activity can also be modulated at the post-translational level by regulation of LPL degradation, LPL transport across the endothelium (He et al., 2018) or through the release of LPL from its endothelial location into the bloodstream (Casanovas et al., 2007; Ricart-Jané et al., 2008), which underscores the importance of LPL binding to HSPG and GPIHBP1 in the stabilization and regulation of functional LPL (Beigneux et al., 2007; He et al., 2018). Importantly, post-translational modifications may also play a role in LPL regulation as proposed for tyrosine nitration in the response to lipopolysaccharide administration (Casanovas et al., 2009b). Contributing to this picture of regulatory mechanisms, recent studies have identified novel proteins involved in the regulation of LPL maturation, stability and activity (Wu et al., 2021). In particular, lipase maturation factor 1 (LMF1) and Sel-1 suppressor of Lin-12-like 1 (SEL1L) are required for proper folding and maturation of LPL protein. On the other hand, angiopoietin-like proteins (ANGPTL) 3, 4 and 8 downregulate LPL activity in a tissue-specific manner, whereas GPIHBP1 may counteract this effect by stabilizing LPL structure. Furthermore, apolipoproteins C-II and A-V activate LPL, whereas apolipoproteins C-I, C-III and E inhibit the enzyme. Overall, clear progress has been made with the discovery of these regulators, but multiple aspects of tissue-specific LPL regulation remain incompletely understood (Wu et al., 2021).

Further expanding the complexity of LPL biology, our previous work revealed that LPL comprises a group of proteoforms with different isoelectric points (pI) both in rat heart (Casanovas et al., 2009a) and human post-heparin plasma (Badia-Villanueva et al., 2014). Knowledge on the molecular origin of LPL proteoforms has thus far been limited to the partial contribution of glycans to LPL pI heterogeneity (Casanovas et al., 2009a). Even more scarce is our knowledge on the regulation and functional significance of LPL proteoforms, which remain completely unexplored. Here, we studied the distribution of LPL proteoforms in different tissues and their regulation under physiological conditions. In addition, we investigated potential differences between proteoforms in their lipolytic activity and heparin-binding affinity as central properties of LPL function.

## Materials and methods

### Animals and samples

Male Wistar rats were purchased from Harlan Interfauna Iberica (Barcelona, Spain) and 15-day-old Wistar rat pups were provided by the animal facility of the Faculty of Biology, Universitat de Barcelona. For the study of LPL proteoforms in rat, different tissues were obtained from 30 adult animals and 40 rat pups, frozen in liquid nitrogen and kept at −80°C until use. To study potential variations in the pattern of LPL proteoforms in response to fasting and refeeding, a different set of animals was distributed into three experimental groups (10 animals/group): control (rats fed *ad libitum*), fasting (rats fasted for 19 h prior to sacrifice) and refeeding (rats fasted for 17 h and subsequently refed for 2 h prior to sacrifice). Rats were sacrificed by decapitation and WAT was extracted, frozen in liquid nitrogen and kept at −80°C until use. Procedures involving rats were approved by the Committee on Animal Bioethics and Care of the Universitat de Barcelona and the Generalitat (Autonomous Regional Government) of Catalonia, Spain (procedure numbers 6429, 6666 and 7440).

WAT from adult cynomolgus monkeys were obtained as a by-product of a study approved by the Generalitat (Autonomous Regional Government) of Catalonia, Spain (procedure number 7231). Cynomolgus monkeys were provided by Harlan Interfauna Iberica (Barcelona, Spain). WAT samples were obtained from 13 adult males, frozen in liquid nitrogen and kept at −80°C until use.

### Heparin-Sepharose chromatography

LPL was partially purified using heparin-Sepharose affinity chromatography, essentially as described elsewhere (Ramirez et al., 1985). Briefly, chromatography was performed at 4°C and at a constant flow rate of 0.25 mL/min. A heparin-Sepharose CL-6B column (0.7 cm × 30 cm) was equilibrated with 10 mM Tris–HCl pH 7.4 buffer containing 30% v/v glycerol and 0.15 M NaCl. All tissues were homogenized 1:5 (w:v) in 10 mM HEPES, 1 mM EDTA, 1 mM DTT, 0.3% w/v sodium deoxycholate, pH 7.5. Tissue homogenate was adjusted to 0.15 M NaCl and loaded into the column. After sample application, the column was washed and LPL was eluted by stepwise increase in NaCl concentration. Fractions were collected throughout the process. Fatty acid-free bovine serum albumin (BSA) was added (1 mg/mL final concentration) to a small aliquot from each fraction to preserve LPL activity (Ramirez et al., 1985). Protein concentration and LPL activity were determined in fractions following standard procedures (Bradford, 1976; Julve et al., 1996). LPL-containing fractions were pooled and frozen at −80°C. For further analysis, proteins were precipitated using trichloroacetic acid followed by acetone washing as described elsewhere (Quero et al., 2004) and redissolved in a buffer appropriate for the analysis.

### LPL activity

LPL activity was determined as described elsewhere (Julve et al., 1996). One unit of lipase activity corresponds to the release of 1 μmol of oleate per minute at pH 8.5°C and 25°C. LPL activity was determined in tissue homogenates and also in fractions collected during the heparin-Sepharose chromatography.

### Two-dimensional electrophoresis (2DE)

After protein precipitation, partially purified LPL was processed as described elsewhere (Casanovas et al., 2009a) and applied to rehydrated immobilized pH-gradient (IPG) strips (11 cm, pH 6–11; GE Healthcare, Uppsala, Sweden) by cup-loading at the cathode. Isoelectric focusing (IEF) was performed at 20°C on IPGphor (GE Healthcare, Uppsala, Sweden) according to the following protocol: linear ramp to 500 V in 1 h, linear ramp to 1,000 V in 1 h, linear ramp to 5,000 V in 1 h and 5,000 V/h up to 25 kV h. After that, focused IPG strips were cut at 7.5 cm from the anode (pH 6) and equilibrated. Equilibrated strips were loaded onto a 9% w/v polyacrylamide gel and sealed using a solution containing 0.5% w/v agarose, 25 mM Tris, 0.1% w/v SDS, 192 mM glycine and bromophenol blue for protein separation in the second dimension by SDS-PAGE. SDS-PAGE was run until the blue dye front reached the bottom of the gel.

### Silver staining

After electrophoresis, proteins were silver-stained using a procedure compatible with mass spectrometry, as previously described (Casanovas et al., 2009a).

### LPL western blot analysis

After electrophoresis, proteins were transferred (1 h 100 V) to a nitrocellulose membrane and LPL was immunodetected by Western blot as described elsewhere (Casanovas et al., 2009a), using monoclonal antibody 5D2 1:2,000 v:v (kind gift of Dr. J. D. Brunzell, University of Washington, Seattle, WA, USA).

### Differential activity-based gel electrophoresis (DABGE)

DABGE probe (NBD, N-(7-Nitrobenz-2-oxa-1,3-diazol-4-il)amine) was prepared as recommended for lipases by Morak et al. (2009). In short, 100 μL of diluent solution (6.25 mg/mL of Triton X-100 in chloroform) and 100 μL of a suicide substrate (0.1 μmol/mL of NBD-D-HP in chloroform) were mixed and dried in a glass tube and subsequently used as the activity-recognition probe. This substrate carries a fluorescent probe and binds covalently to the enzyme when hydrolyzed.

DABGE analysis had to be adapted in order to i) avoid the cross-reactivity of BSA (usually added to stabilize LPL activity in fractions collected during heparin-Sepharose chromatography) with the suicide substrate (Kim et al., 2013) and ii) optimize the incubation conditions. As a result, partially purified LPL was immediately mixed after elution from heparin-Sepharose chromatography (1.35 mL) with DABGE probe (final concentration 0.075 μmol/mL) and incubated for 1 h at 25°C in the dark.

After incubation, samples were precipitated and analyzed by 2DE as described above. After 2DE, the gel was analyzed using a fluorescence scanner (Typhoon FLA 9500; GE Healthcare, Uppsala, Sweden) for detection of proteins with lipolytic (or esterolytic) activity.

### Difference gel electrophoresis (DIGE)

After protein precipitation, partially purified LPL was incubated with 30 μL of DIGE buffer (7 M urea, 2 M thiourea, 2% w/v CHAPS, Tris-HCl 20 mM, pH 8.5) and each sample was mixed with one fluorophore from a CyDye DIGE Fluor minimal labeling kit (GE Healthcare, Uppsala, Sweden) following the manufacturer’s instructions. After incubation for 30 min in a cold and dark chamber, 1 μL of lysine solution (10 mM) was added, mixed and incubated for 10 min in the same conditions. Finally, 32 μL of DIGE buffer supplemented with 1% v/v IPG Buffer pH 6–11 and 18 mM DTT, were added and all samples were mixed together and analyzed by 2DE as described above in the same gel. After electrophoresis, gels were analyzed using a fluorescence scanner (Typhoon FLA 9500; GE Healthcare, Uppsala, Sweden) at different wavelengths (specific for each fluorophore) and images were overlapped for comparison.

### Quantification and pI of LPL proteoforms

Quantification of LPL proteoforms was done on images of LPL 2DE Western blot. Specifically, all spots were quantified by densitometry (Multi Gauge, Fujifilm) and the abundance of each proteoform was calculated relative to the total LPL content in the sample. Each sample was analyzed by 2DE Western blot in 2–4 technical replicates (depending on sample availability), as specified in the figure legends. In turn, the measurement of proteoform abundance in each 2DE Western blot was obtained as the average of at least two different exposures. On the other hand, the pI of each proteoform was determined based on the linear pH gradient of the IPG strips, by measuring the distance between the proteoform and the anodic edge (pH 6) of the gel. Having calculated the relative abundance and pI for each proteoform, we depicted a representative pattern of LPL proteoforms for each tissue and condition. LPL proteoform patterns from different tissues and conditions were compared as described below.

### In-gel digestion

The spots of interest were excised from silver-stained gels and subjected to in-gel digestion with trypsin (Promega, Madison, WI, USA) using a Digest ProMS (Intavis Bioanalytical Instruments AG, Koeln, Germany) as previously described (Casanovas et al., 2009a).

### Protein identification by mass spectrometry

Tryptic digests from LPL gel spots were analyzed by liquid chromatography coupled to mass spectrometry (LC-MS/MS) using an LTQ XL Orbitrap (ThermoFisher, San Jose, CA, USA) equipped with a nanoESI ion source. A volume of 20 μL from each sample was loaded into the chromatographic system consisting of a C18 preconcentration cartridge (Agilent Technologies, Barcelona, Spain) connected to a 15 cm long, 100 μm i.d. C18 column (Nikkyo Technos Co.). The separation was done at 0.4 μL/min in a 30-min acetonitrile gradient from 3% to 40% (solvent A: 0.1% formic acid, solvent B: acetonitrile 0.1% formic acid). The HPLC system comprised an Agilent 1200 capillary nano pump, a binary pump, a thermostated micro injector and a micro switch valve. The LTQ XL Orbitrap was operated in the positive ion mode with a spray voltage of 1.8 kV. The spectrometric analysis was performed in a data-dependent mode, acquiring a full scan followed by 10 MS/MS scans of the 10 most intense signals detected in the MS scan. The full MS (range 300–1,800) was acquired in the Orbitrap with a resolution of 60,000. The MS/MS spectra were acquired in the linear ion-trap using CID for fragmentation. To avoid the redundant selection of precursor ions, dynamic exclusion was set to 1 with an exclusion time of 30 s. The fragmentation spectra were searched using SEQUEST (Proteome Discoverer v1.4, ThermoFisher) with the following parameters: peptide mass tolerance 20 ppm, fragment tolerance 0.6 Da, enzyme set as trypsin and allowance of up to two missed cleavages, cysteine carbamidomethylation as a fixed modification and oxidation of methionine as a dynamic modification. The database used for searching was *Macaca fascicularis*. The results were filtered by peptide rank 1, peptide confidence high and two peptides per protein. The mass spectrometry proteomics data have been deposited to the ProteomeXchange Consortium via the PRIDE (Perez-Riverol et al., 2022) partner repository with the dataset identifier PXD043704.

### Statistical analysis

For the comparison of LPL proteoform patterns from different tissues or conditions the results were analyzed using “within-subject” confidence intervals (CIs) (Loftus and Masson, 1994) for one-way ANOVA, where the ANOVA assumptions were fulfilled: equality of variances (Bartlett’s test) and normal distribution (Shapiro-Wilk test). Inference with CIs is usually accomplished merely by determining whether the CI overlaps between individual means of the levels of the factor. In our approach, we define the same proteoform by comparing the CI of the means of the proteoform pI (factor) in different tissues or conditions (level). If the CI of the proteoform pI overlapped, they were considered the same proteoform in the tissues or conditions compared. Statistical comparison of proteoform relative abundance was performed by Student’s t-test or by one-way ANOVA and *post hoc* Tukey test. Statistical comparison of LPL activity in tissues was performed by Student’s t-test. Differences were significant when *p* < 0.05.

## Results

### LPL proteoforms are differentially distributed in rat tissues

**FIGURE 1:**
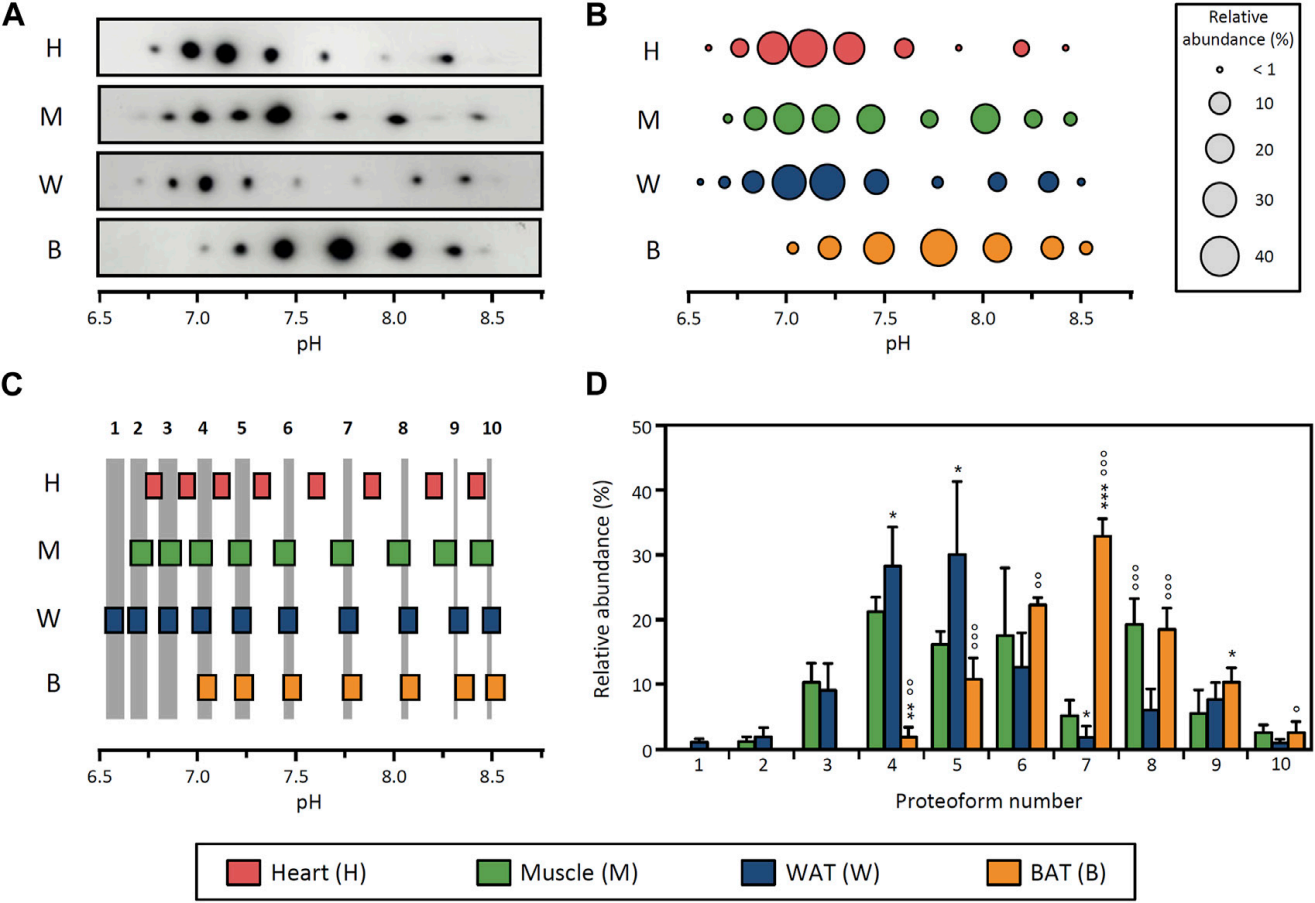
Distribution of LPL proteoforms in rat tissues. **(A)** 2DE Western blot against LPL of partially purified LPL from rat heart, skeletal muscle, epididymal WAT and BAT. **(B)** Representative pattern of LPL proteoforms that shows the pI and relative abundance (circle size) of each proteoform. **(C)** Comparison of pI values of LPL proteoforms across tissues. LPL proteoforms with overlapping 95% CI for pI values were regarded as the same proteoform. Grey stripes indicate the pH range with overlapping 95% CI across grouped tissues. Numbers at the top of the grey stripes indicate the proteoform number. **(D)** Relative abundance of LPL proteoforms. Proteoforms are numbered as indicated in **(C)**. The results are expressed as the mean ± SD (n = 2–4). Statistical comparison by one-way ANOVA and *post hoc* Tukey test. *p < 0.05; **p < 0.01; ***p < 0.001, compared with skeletal muscle; ○ *p* < 0.05; ○○ *p* < 0.01; ○○○ *p* < 0.001, compared with WAT.

To study the distribution of LPL proteoforms in different tissues, we obtained heart, skeletal muscle, epididymal WAT and BAT from adult rats under basal conditions (i.e., resting and fed *ad libitum*). LPL was partially purified from tissue homogenates using heparin-Sepharose affinity chromatography and subsequently analyzed by 2DE followed by Western blot (2DE Western blot) against LPL (Supplementary Figure S1). All tissues exhibit between 7 and 10 LPL proteoforms with pI values ranging from 6.5 to 8.5 (Figure 1A). For comparative purposes, for each tissue we determined the pI and relative abundance of each proteoform and depicted a representative proteoform pattern (Figure 1B).

To evaluate potential differences between patterns of LPL proteoforms, we devised a strategy comprising two steps. First, we calculated the 95% CI for the pI value of each proteoform and compared the values obtained across tissues (Figure 1C). LPL proteoforms from different tissues with overlapping 95% CI were considered to have the same pI and were therefore regarded as the same proteoform. Based on this approach, two groups of tissues could be distinguished: a group comprising skeletal muscle, WAT and BAT (where all proteoforms showed overlapping 95% CI for pI values) and a second group comprising heart only (Figure 1C). We note that, despite the overlap of some LPL proteoforms from heart and muscle, muscle was ascribed to the first group based on the higher number of overlapping proteoforms (5 vs. 9). In the second step of the comparison of proteoform patterns, we contrasted the relative abundance of proteoforms between tissues within the same group. This analysis showed differences in the relative abundance of LPL proteoforms between muscle, WAT and BAT (Figure 1D). Specifically, LPL proteoforms in BAT are generally more abundant in the pH range ≥7.5 (proteoforms 7–10 represent 65% of total LPL content), whereas WAT LPL proteoforms are more abundant at pH ≤ 7.5 (proteoforms 1–6 represent 83% of total LPL content). In contrast, the abundance of LPL proteoforms in skeletal muscle is more evenly distributed throughout a wider pH range (6.7–8.5).

These results demonstrate that the pattern of LPL proteoforms differs between tissues either by differences in their pI distribution or relative abundance. Importantly, in light of the tissue-specific regulation of the enzyme, the fact that tissues display different proteoform patterns suggests that LPL proteoforms may have different functional properties.

### Tissue-specific patterns of LPL proteoforms are also present in rat pups

To determine whether the tissue-specific patterns of LPL proteoforms observed in adult rats were also present in an independent physiological condition, we analyzed tissues from 15-day-old rat pups. For this comparison, we analyzed heart and BAT as these tissues exhibit remarkably different LPL proteoform patterns in adult rats (Figure 1). Besides, LPL activity in heart and BAT is similar between 15-day-old and adult rats (Galan et al., 1993), and therefore only minimal LPL regulation could be expected.

**FIGURE 2:**
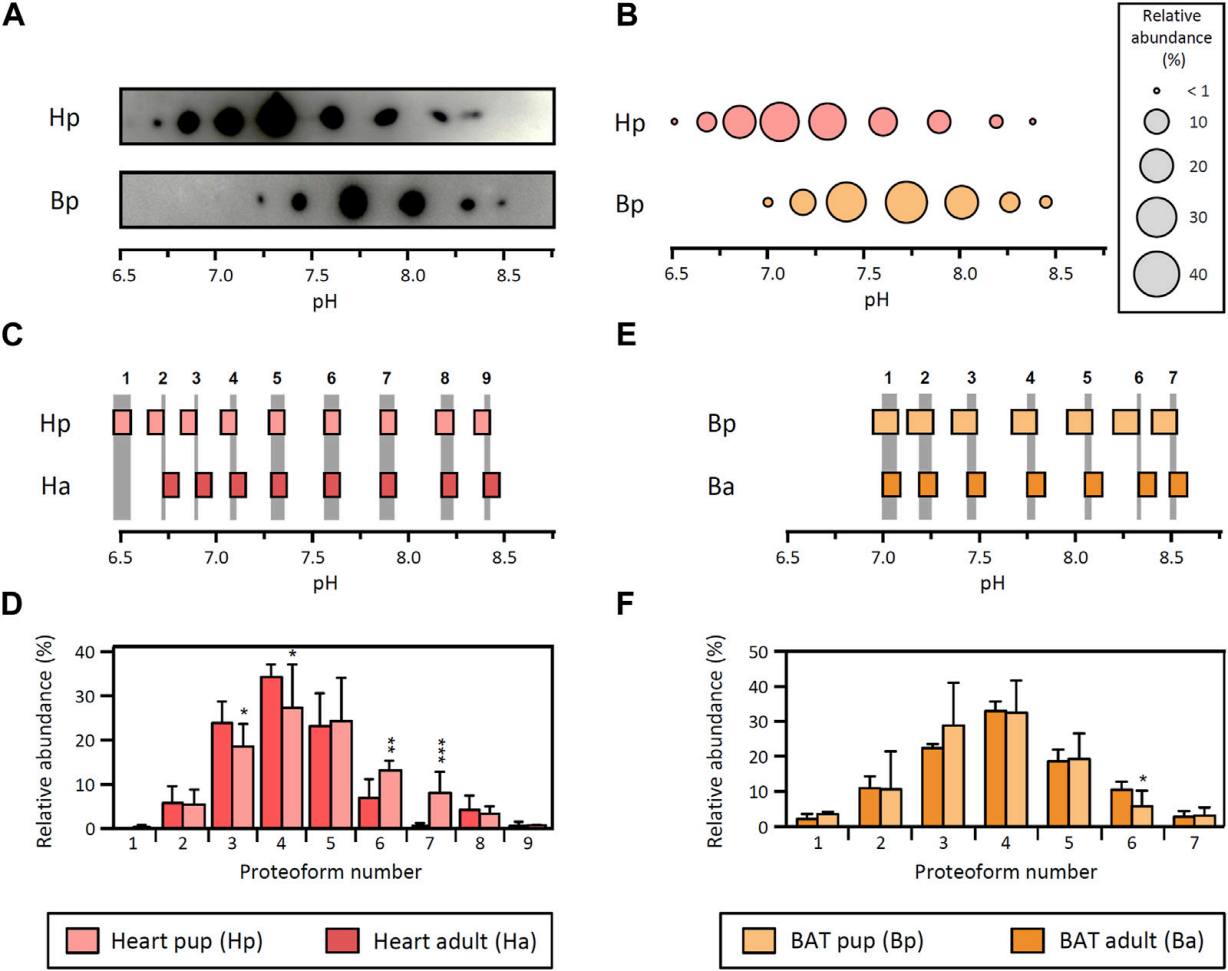
LPL proteoforms in tissues from rat pups. **(A)** 2DE Western blot against LPL of partially purified LPL from heart and BAT from 15-day-old rat pups. **(B)** Representative pattern of LPL proteoforms that shows the pI and relative abundance (circle size) of each proteoform. **(C–F)** Comparison between adult and pup LPL proteoforms from heart **(C,D)** and BAT **(E,F)**. Data from adult heart and BAT are as in Figure 1. **(C,E)** Comparison of 95% CI for pI values from heart **(C)** and BAT **(E)**. LPL proteoforms with overlapping 95% CI for pI values were regarded as the same proteoform. Grey stripes indicate the pH range with overlapping 95% CI. Numbers at the top of the grey stripes indicate the proteoform number. **(D,F)** Relative abundance of LPL proteoforms from heart **(D)** and BAT **(F)**. Proteoforms are numbered as indicated in **(C)** for heart and **(E)** for BAT. The results are expressed as the mean ± SD (n = 3). Statistical comparison by Student’s t-test. *p < 0.05; **p < 0.01; ***p < 0.001.

LPL proteoforms from heart (9 proteoforms) and BAT (7 proteoforms) in rat pups are distributed in the pH range 6.5–8.5 (Figures 2A, B). Comparison of these patterns shows that the 95% CI for pI values of heart and BAT from rat pups do not overlap (comparison not shown), leading to the conclusion that LPL proteoforms from both tissues have a different pI and, consequently, that their relative abundance cannot be compared, which is consistent with the result observed in adult rats.

Next, the pI patterns obtained in heart and BAT from rat pups were compared with their counterparts from adult rats. Notably, heart LPL proteoforms from rat pups exhibit the same number of proteoforms and pI (overlapping 95% CI for pI values) as adult rats (Figure 2C). The comparison of proteoform abundance showed minor differences between pups and adults, with proteoforms 3 and 4 showing a higher abundance in adults and proteoforms 6 and 7 showing a higher abundance in pups (Figure 2D). On the other hand, the pattern of LPL proteoforms in BAT from pups is virtually identical to that of adult rats, except for a slight difference in the relative abundance of proteoform 6 (Figures 2E, F).

Overall, the results obtained in pups match the differences between tissues observed in adult rats and substantiate the existence of tissue-specific patterns of LPL proteoforms. Furthermore, tissue-specific patterns of LPL proteoforms seem to be conserved at different developmental stages and support the premise that different LPL proteoforms may exhibit distinct functional properties.

### The pattern of LPL proteoforms in WAT is modified with fasting and restored upon refeeding

To determine whether LPL proteoforms have distinct functional properties, we investigated potential changes in the proteoform pattern in a physiological condition that entails tissue-specific regulation of LPL activity. We hypothesized that, if LPL proteoforms are functionally diverse, the pattern of LPL proteoforms would change when LPL activity is highly regulated. In contrast, if all proteoforms are identical from the functional point of view, one would expect only minor changes (if any) in the pattern of LPL proteoforms upon tissue-specific regulation of LPL activity.

**FIGURE 3:**
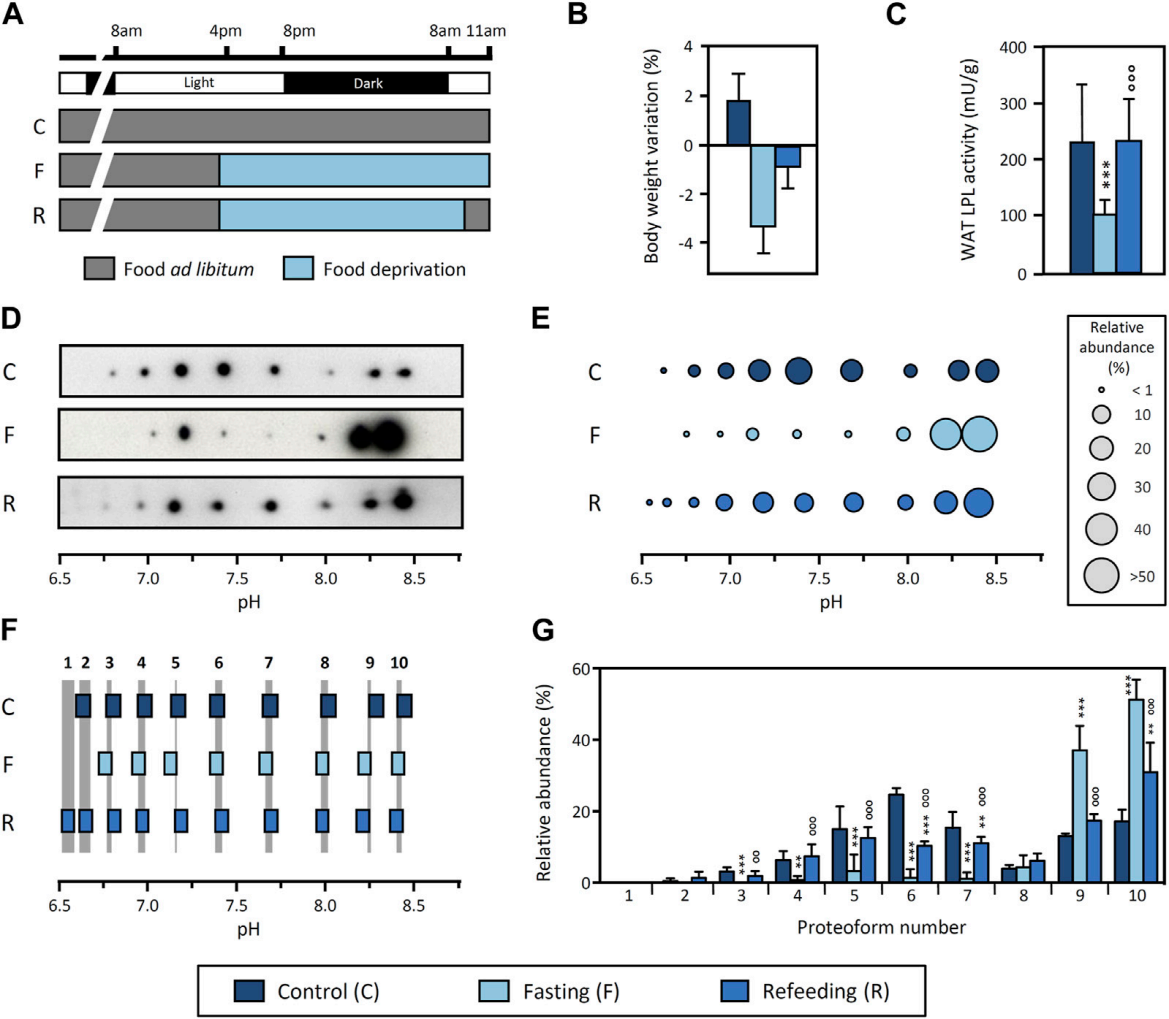
Regulation of LPL proteoforms from rat WAT in response to fasting and refeeding. **(A)** Experimental design. Food was removed 19 h before sacrifice from both the “Fasting” and “Refeeding” groups. Two hours before sacrifice, food was reintroduced in the “Refeeding” group. “Control” group was fed *ad libitum* throughout the experiment. Body weight variation **(B)** and LPL activity in epididymal WAT **(C)** in response to fasting and refeeding. **(D)** 2DE Western blot against LPL of partially purified LPL from epididymal WAT. **(E)** Representative pattern of LPL proteoforms that shows the pI and relative abundance (circle size) of each proteoform. **(F)** Comparison of pI values of LPL proteoforms. LPL proteoforms with overlapping 95% CI for pI values were regarded as the same proteoform. Grey stripes indicate the pH range with overlapping 95% CI. Numbers at the top of the grey stripes indicate the proteoform number. **(G)** Relative abundance of LPL proteoforms. Proteoforms are numbered as indicated in **(F)**. The results are expressed as the mean ± SD (n = 2–4). Statistical comparison of proteoform relative abundance by one-way ANOVA and *post hoc* Tukey test or Student’s t-test for LPL activity. **p < 0.01; ***p < 0.001, compared with Control group; ○○ *p* < 0.01; ○○○ *p* < 0.001, compared with Fasting group.

To address this hypothesis, we analyzed the pattern of LPL proteoforms in response to fasting and refeeding, as this physiological context causes a pronounced downregulation of WAT LPL activity during fasting and a recovery upon refeeding (Doolittle et al., 1990; Bergö et al., 1996a). We conducted an experiment with three experimental groups: i) control (rats fed *ad libitum*), ii) fasting (rats fasted for 19 h prior to sacrifice) and iii) refeeding (rats fasted for 17 h and subsequently refed for 2 h prior to sacrifice) (Figure 3A). To validate the experimental approach and confirm the expected physiological response, we analyzed body weight and LPL activity in tissues. Our results show a decrease in body weight with fasting and recovery upon refeeding (Figure 3B). In addition, epididymal WAT LPL activity is drastically decreased during fasting and restored upon refeeding (Figure 3C), whereas LPL activity in heart is upregulated during fasting (control: 294 ± 23 mU/g tissue vs. fasting: 453 ± 17 mU/g tissue, *p*-value <0.001) and subsequently reduced upon refeeding (fasting 453 ± 17 mU/g tissue vs. refeeding: 290 ± 18 mU/g tissue, *p*-value <0.001). Overall, these results are consistent with previous reports (Quig et al., 1983; Doolittle et al., 1990; Bergö et al., 1996a) and demonstrate that the experimental conditions reproduced the expected response.

Next, we investigated the pattern of LPL proteoforms in epididymal WAT under these conditions. The results show 9, 8 and 10 proteoforms in the control, fasting and refeeding group, respectively, within a pH range from 6.5 to 8.4 (Figures 3D, E). Comparison of the 95% CI for pI values indicates that all patterns contain the same proteoforms, regardless of the physiological condition (Figure 3F). However, there are important differences in the relative abundance of LPL proteoforms between conditions (Figure 3G). Remarkably, fasting causes an increase in the abundance of basic proteoforms (pH ≥ 8.1) as compared to the control group. In fact, two proteoforms represent >80% of the total LPL content during fasting. Importantly, refeeding reverts this change and shows an intermediate proteoform pattern as a transition between the fasting and control conditions (Figures 3D,E,G).

Because this comparison was made using LPL proteoform patterns obtained from separate gels for each condition, we conducted an additional analysis using DIGE to validate that LPL proteoforms from all conditions have the same pI. In DIGE, each sample is initially tagged with a unique fluorescent dye and samples are subsequently combined and analyzed in a single 2DE. Each sample can be visualized separately by fluorescence scanning or superimposed for comparison between samples (a protein present in more than one condition shows “overlapping color”). The results of this analysis show the overlap of LPL proteoforms from control, fasting and refeeding groups and therefore confirm that the pI of LPL proteoforms does not differ between conditions (Supplementary Figure S2). Notably, this result also corroborates our general strategy for pI alignment and comparison of proteoform patterns from separate gels (Figures 3D–F).

We conclude that the pattern of LPL proteoforms in WAT is regulated by fasting and restored upon refeeding, coinciding with abrupt changes in LPL activity. These findings indicate that LPL proteoforms are functionally diverse as the transient downregulation of LPL activity in tissue is accompanied by a reversible reconfiguration of the LPL proteoform pattern.

### All LPL proteoforms from WAT are enzymatically active

**FIGURE 4:**
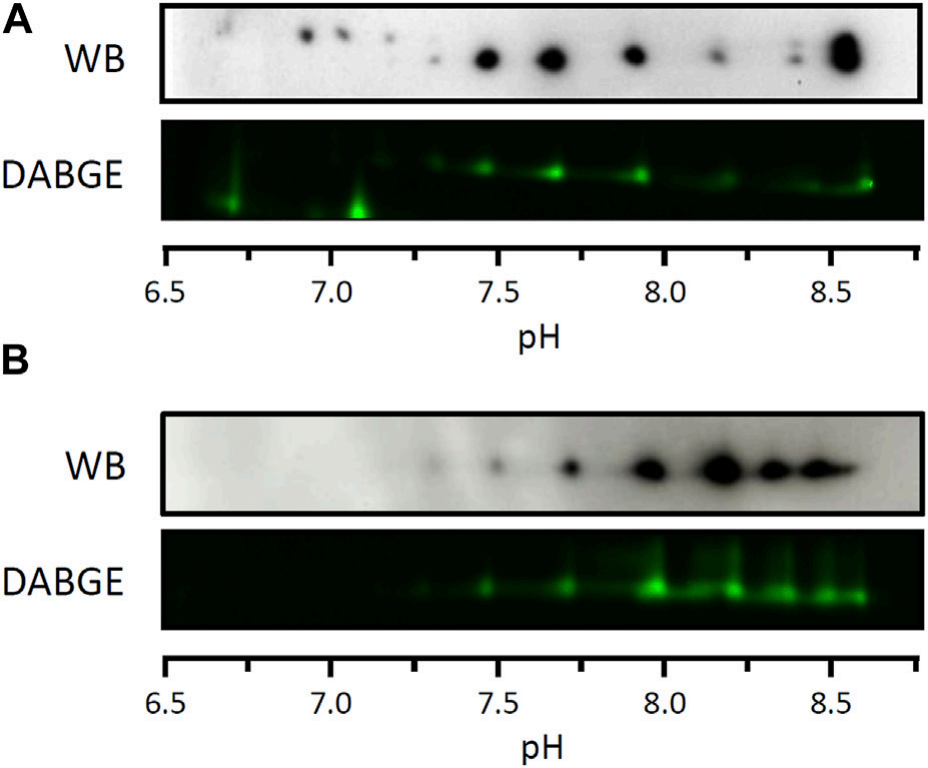
Lipolytic activity of LPL proteoforms from WAT. Lipolytic activity of individual proteoforms assessed by differential activity-based gel electrophoresis (DAGBE) of partially purified LPL from rat **(A)** and cynomolgus monkey **(B)** WAT. 2DE Western blots against LPL of equivalent samples are shown on top of the DABGE images.

To assess potential differences in functional properties between LPL proteoforms, we first investigated whether all proteoforms exhibit lipolytic activity. To this end, given the lack of methods to isolate individual LPL proteoforms while preserving their activity, we analyzed partially purified LPL using DABGE. This method was originally developed to identify differential proteins with lipolytic or esterolytic activity between samples (Morak et al., 2009) and uses a substrate with a fluorescent probe that, when hydrolyzed, binds covalently to the enzyme. In our study, we adapted the use of this substrate to determine whether all LPL proteoforms show lipolytic activity. The results obtained revealed that all LPL proteoforms from rat epididymal WAT are catalytically active, as all proteoforms detected by Western blot also exhibit fluorescence when incubated with the DABGE substrate (Figure 4A).

In order to substantiate this result, we repeated this assay in another species. In particular, we analyzed LPL from cynomolgus monkey visceral WAT because i) the LPL sequence in this species is highly conserved in relation to the human sequence as compared to rat (Holmes, 2011) and ii) large(r) amounts of tissue can be obtained from individual subjects. LPL from cynomolgus monkey visceral WAT was partially purified as described above for rat tissues and subsequently analyzed by 2DE Western blot (Supplementary Figure S3). Since the antibody used in the Western blot (5D2) has not previously been used to detect cynomolgus monkey LPL, and given the non-specificity of other antibodies against LPL previously used in the literature (Casanovas et al., 2008), we aimed to confirm the identity of LPL proteoforms using mass spectrometry. For this, the sample was analyzed by 2DE followed by silver staining and the spots of interest were excised for protein identification by LC-MS/MS (Supplementary Figure S3). Following the unambiguous identification of LPL from cynomolgus monkey visceral WAT by MS (Supplementary Table S1), the sample was analyzed by DABGE. The results show that all LPL proteoforms detected by Western blot are catalytically active (Figure 4B) as previously observed for LPL from rat WAT (Figure 4A).

Taken together, these results indicate that all LPL proteoforms from both rat and cynomolgus monkey WAT exhibit lipolytic activity. We note, however, that the approach used here is not suitable for a quantitative analysis and, therefore, potential differences in the catalytic rate between proteoforms cannot be ruled out.

### LPL proteoforms have similar heparin-binding affinity

**FIGURE 5:**
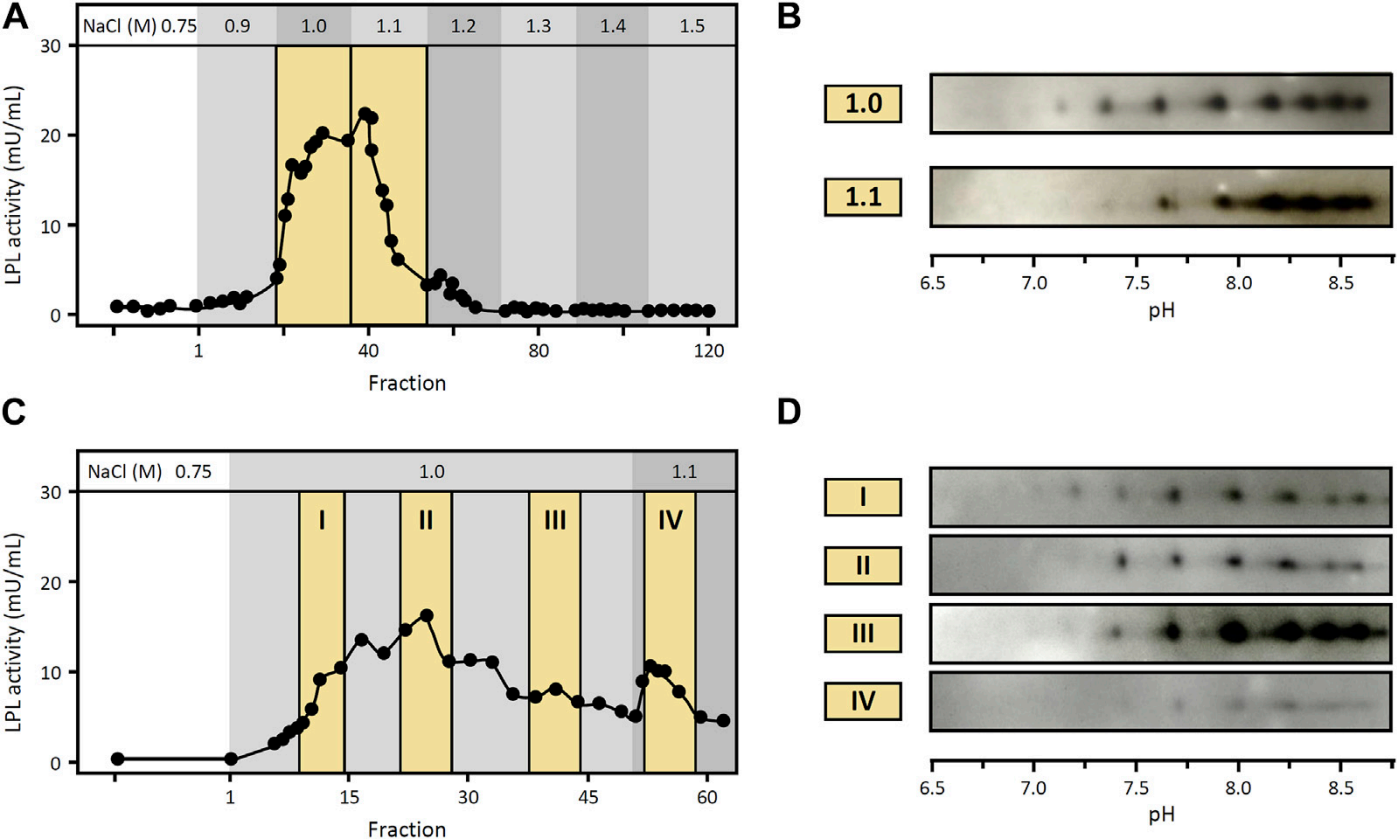
Heparin-binding affinity of LPL proteoforms from WAT. Heparin-Sepharose affinity chromatography of LPL from cynomolgus monkey WAT using modified elution procedures **(A,C)**. **(A)** LPL activity in fractions collected after stepwise increase in NaCl concentration as indicated at the top. Fractions are numbered starting from the first fraction of the elution step. Fractions pooled and analyzed by 2DE Western blot are highlighted with a yellow background. **(B)** 2DE Western blot against LPL of fractions collected at 1.0 M and 1.1 M NaCl, as indicated in **(A)**. **(C)** LPL activity in fractions collected with 1.0 M NaCl as elution buffer followed by 1.1 M NaCl as indicated at the top. Fractions are numbered starting from the first fraction of the elution step. Fractions pooled and analyzed by 2DE Western blot are highlighted with a yellow background. **(D)** 2DE Western blot against LPL of fractions collected at 1.0 M and 1.1 M NaCl, as indicated in **(C)**.

Given the importance of LPL binding to HSPG in the stabilization and regulation of the functional enzyme, we investigated potential differences in heparin-binding affinity between LPL proteoforms. To this end, we used heparin-Sepharose affinity chromatography with modified stepwise elution procedures to assess whether different LPL proteoforms from cynomolgus monkey visceral WAT can be eluted separately. More specifically, the standard protocol for heparin-Sepharose affinity chromatography used in the experiments above comprised a pre-elution washing step with 0.75 M NaCl followed by an elution step with 1.5 M NaCl (Supplementary Figures S1A, S3). Here, we first modified the elution procedure to include, after the washing step with 0.75 M NaCl, a 0.1 M stepwise increase in NaCl concentration from 0.9 to 1.5 M (Figure 5A). LPL activity was observed in fractions collected during elution with 1.0 and 1.1 M NaCl. Analysis by 2DE Western blot against LPL of fractions collected during elution with 1.0 and 1.1 M NaCl showed almost identical patterns of LPL proteoforms (Figure 5B), suggesting co-elution of LPL proteoforms.

However, in this first procedure i) all fractions eluted with 1.0 M NaCl were pooled for 2DE Western blot analysis and ii) elution with 1.0 and 1.1 M NaCl yielded a single peak of LPL activity (Figure 5A). Consequently, we conducted another experiment extending the elution step with 1.0 M NaCl in order to i) separately analyze fractions collected at different times of this elution step and ii) assure that elution with 1.0 M NaCl was complete prior to the switch to 1.1 M NaCl. Specifically, after the washing step with 0.75 M NaCl, elution was carried out with 1.0 M NaCl for 6 h (3-fold longer than the previous chromatography) followed by a final elution step with 1.1 M NaCl (Figure 5C). LPL activity was detected in fractions collected during these elution steps and fractions from different elution times were selected for subsequent analysis by 2DE Western blot. Again, the results of 2DE Western blot against LPL showed the same pattern of LPL proteoforms in all fractions analyzed (Figure 5D).

Overall, using these approaches, we observed co-elution of LPL proteoforms, which suggests that LPL proteoforms have similar heparin-binding affinity.

## Discussion

In recent decades, advances in the fields of proteomics and biological mass spectrometry have revealed that proteome complexity is much greater than could be inferred from genome sequencing (Aebersold et al., 2018). In this sense, it is now apparent that the original axiom “one gene = one protein” has become in most instances obsolete, as multiple proteoforms can derive from a single gene (Schlüter et al., 2009; Smith et al., 2013). In fact, there are multiple examples of proteins with a prominent role in physiology and disease that were traditionally considered as a single protein and have subsequently been revisited as a group of proteoforms (Fojo et al., 1986; Selkirk et al., 1996; Natale et al., 2010; Ruzo et al., 2015). LPL is no exception to this common trait. Indeed, early work from Soteriou and Cryer resolved LPL with different pI values in 2DE gels (Soteriou and Cryer, 1993) but tentatively attributed this to the potential binding of ampholytes to LPL in IEF, on the basis of a previous study that suggested ampholyte binding to bovine LPL when IEF was conducted under native conditions (Bengtsson and Olivecrona, 1977). Subsequent studies, carried out in our group using denaturing conditions and IPG strips for IEF, ruled out the possibility of ampholite binding and, in combination with Western blot and MS, demonstrated the existence of LPL proteoforms in rat heart, bovine milk and human post-heparin plasma (Casanovas et al., 2009a; Badia-Villanueva et al., 2014). Extending these findings, in the present study we report the existence of LPL proteoforms in different rat tissues and WAT of cynomolgus monkey, which further denotes the ubiquitous nature of LPL proteoforms in mammals and suggests conserved features in LPL proteoforms across species.

In general terms, studies on the characterization of proteoforms have mainly focused on elucidating differences between proteoforms at the molecular level whereas the potential differences in their regulation and function remain largely unexplored. In the case of LPL proteoforms, the molecular origin of pI heterogeneity remains poorly characterized but is likely related to PTMs. In this sense, we have previously described the partial contribution of glycosylation to the pI heterogeneity of LPL and demonstrated that protein phosphorylation does not contribute to LPL pI heterogeneity (Casanovas et al., 2009a). In another study, we identified nitrated tyrosine residues in rat LPL in response to LPS challenge (Casanovas et al., 2009b), demonstrating that LPL can undergo other PTMs *in vivo*. Hence, tyrosine nitration or other PTMs not yet characterized could potentially contribute to LPL pI heterogeneity. In contrast, the regulation and functional significance of LPL proteoforms have not been studied thus far. Here, we combined different approaches to explore potential differences in the regulation and function of LPL proteoforms. First, on the basis of the well-described tissue-specific regulation of LPL, we investigated the distribution of LPL proteoforms in several rat tissues under basal conditions and observed different proteoform patterns in LPL from heart, skeletal muscle, epididymal WAT and BAT. This finding is consistent with previous studies reporting that LPL from different tissues has different properties in terms of enzyme kinetics, thermolability, specific activity and immunoreactivity, which suggested the existence of tissue-specific variants of the enzyme (Fielding, 1976; Ben-Zeev et al., 1983; Soteriou and Cryer, 1993; Soteriou and Cryer, 1994). Importantly, the fact that tissues with different LPL regulation exhibit distinct patterns of LPL proteoforms suggests that different LPL proteoforms may have distinct functional properties. To contrast this possibility, we investigated whether the pattern of LPL proteoforms in individual tissues changes under different conditions. Specifically, we first compared the pattern of LPL proteoforms in heart and BAT between adult and 15-day-old rat pups, a developmental stage that coincides with nutritional and hormonal changes at the onset of the weaning period, when pups start eating solid food (Redman and Sweney, 1976; Henning, 1978). Notably, these two conditions were selected because they show comparable levels of LPL activity both in heart and BAT (Galan et al., 1993) and therefore minimal LPL regulation can be expected. This comparison yielded virtually the same (tissue-specific) patterns of LPL proteoforms between adults and pups, indicating that in the absence of LPL regulation the pattern of LPL proteoforms remains unchanged.

We also investigated potential changes in the pattern of LPL proteoforms in physiological conditions in which LPL activity is highly regulated, that is, fasting and refeeding. Indeed, it has been widely reported that, during fasting, LPL activity is markedly decreased in WAT whereas this tissue-specific regulation is reverted upon refeeding (Doolittle et al., 1990; Bergö et al., 1996a). Importantly, the decrease in WAT LPL activity during short-term fasting (i.e., ≤24 h) and subsequent refeeding occurs essentially without changes in the levels of LPL mRNA, the relative rate of LPL biosynthesis or the levels of LPL protein, indicating that LPL activity is regulated at the post-translational level (Doolittle et al., 1990; Erskine et al., 1994; Bergö et al., 1996a; Lee et al., 1998; Bergö et al., 2002). In contrast, when fasting is prolonged, pre-translational regulation also intervenes as evidenced by a decrease in the levels of LPL mRNA, the relative rate of LPL biosynthesis and the levels of LPL protein after 3 days of fasting (Bergö et al., 1996a; Lee et al., 1998). In this study, rats were fasted for 19 h in order to investigate whether the pattern of LPL proteoforms changes when LPL undergoes post-translational regulation. Our results demonstrate that the pattern of LPL proteoforms in WAT is regulated by fasting and restored upon refeeding, coinciding with pronounced changes in LPL activity. Notably, the concomitance between the transient downregulation of LPL activity and the reversible reconfiguration of the LPL proteoform pattern denotes the functional diversity of LPL proteoforms. In fact, previous studies have reported that LPL can exist in at least two forms with different functional properties in terms of activity and heparin-binding affinity. More specifically, these studies have shown that, in the fed state, most LPL is present in the active form with high heparin-binding affinity, whereas inactive LPL with low-heparin binding affinity is the dominant form during fasting (Bergö et al., 1996b). These two forms can be separated by heparin-Sepharose chromatography and elution at different NaCl concentrations. In further detail, inactive LPL is eluted at 0.6 M NaCl, whereas active LPL can be eluted at concentrations ≥1 M NaCl (Bergö et al., 1996b). Here, we partially purified LPL that was still bound to heparin after a 0.75 M NaCl wash and, hence, should be active and have high heparin-binding affinity. Our results show that, besides the two previously reported forms of LPL, a group of proteoforms exists within the LPL form with high heparin-binding affinity, further extending the diversity of LPL forms. Importantly, we did not detect LPL in fractions collected in the 0.75 M NaCl wash (not shown) but, if present, in our experimental approach we would have not been able to capture and further characterize the LPL form with low heparin-binding affinity. We note that if the LPL form with low heparin-binding affinity was present in the tissues studied, the range of LPL proteoforms would be further extended.

To assess potential differences in functional properties between the detected LPL proteoforms, we investigated whether all proteoforms exhibit lipolytic activity and their heparin-binding affinity. Our results showed that all proteoforms present lipolytic activity and similar heparin-binding affinity. However, the experimental approaches used here are not suitable for detecting potential differences between active proteoforms in terms of catalytic rate. On the other hand, several proteins have been reported to play a role in LPL regulation, including GPIHBP1, which is involved in the translocation and binding of functional LPL to the endothelial lumen. Hence, potential differences between LPL proteoforms in the interaction with GPIHBP1 cannot be ruled out and should be considered in future studies. Meanwhile, other regulatory proteins have been reported to play a role in LPL regulation during feeding and fasting. Specifically, ANGPTL4 suppresses LPL activity in WAT during fasting (Dijk et al., 2016) whereas ANGPTL8 has been reported to inhibit ANGPTL4, thus promoting LPL activity in WAT during feeding (Oldoni et al., 2020). In addition, GPIHBP1 protects LPL from the inhibitory effects of ANGPTLs at the endothelium, although recent studies reported that both ANGPTL4 and the ANGPTL3-8 complex may dissociate LPL from GPIHBP1 and inactivate LPL (Shetty et al., 2020). We speculate that nutritional cues that regulate WAT LPL activity in response to fasting and refeeding (Kroupa et al., 2012) could potentially be involved in the regulation of the LPL proteoform pattern either directly or by means of regulatory proteins such as ANGPTL4 (Kroupa et al., 2012).

Taken together, these findings illustrate the complexity of LPL regulation during feeding and fasting and underscore the need to consider all elements involved for a complete understanding of LPL regulation. In this context, our work reveals an additional level of complexity as LPL undergoes proteoform-specific regulation in these physiological conditions, which inherently indicates the functional diversity of LPL proteoforms. Future studies should therefore investigate other functional aspects of individual LPL proteoforms such as their interaction with regulatory proteins or potential differences in their specific activity. In addition, the existence of LPL proteoforms in humans (Badia-Villanueva et al., 2014) and the functional regulation of LPL proteoforms in physiological conditions described here, open the door to further research aimed at investigating the regulation of LPL proteoforms in pathological conditions in humans, as well as their potential impact on pharmacological treatments that modulate LPL activity. Finally, from a broader perspective, our work argues for the need to characterize proteome diversity to advance our understanding of biochemical, physiological and pathological processes, which is aligned with recent initiatives proposed to systematically map all human proteoforms (Smith et al., 2021).

## Data Availability

The datasets presented in this study can be found in online repositories. The names of the repository/repositories and accession number(s) can be found in the article/Supplementary Material.
