# Supplementary material for: The response to fasting and refeeding reveals functional regulation of lipoprotein lipase proteoforms
Source: Front Physiol. 2023 Oct 16;14:1271149. doi: 10.3389/fphys.2023.1271149 (PMC10617031; doi:10.3389/fphys.2023.1271149)
Supplement: Supplementary file 1 [file Table1.pdf]

Carulla et al.
Table S1. Identification of proteins from Figure S3 by LC-MS/MS.

Spot 1

Accession	Description	Score	Coverage	# Proteins	# Unique Peptides	# Peptides	# PSMs	# AAs	MW [kDa]	calc. pI						
G7PUP9	Cytokeratin-10 (Fragment) OS=Macaca fascicularis GN=EGM_07881 PE=3 SV=1 - [G7PUP9_MACFA]	14.12	9.91	1	6	6	6	575	57.8	5.11						
G7PID3	Cytokeratin-2e OS=Macaca fascicularis GN=EGM_03270 PE=3 SV=1 - [G7PID3_MACFA]	13.04	8.42	1	5	5	5	641	65.5	8.02						
Q95KN9	Lipoprotein lipase OS=Macaca fascicularis PE=2 SV=1 - [Q95KN9_MACFA]	9.55	9.13	2	3	3	3	449	50.6	8.57						
	A9	Sequence	# PSMs	# Proteins	# Protein Groups	Protein Group Accessions	Modifications	ΔCn	q-Value	PEP	XCorr	Charge	MH+ [Da]	ΔM [ppm]	RT [min]	# Missed Cleavages
	High	ITGLDPAGPNFEYAEAPSR	1	2	1	Q95KN9		0.0000	0	0.5609	4.37	2	2004.96855	1.15	28.80	0
	High	AQQHYPVSAGYTK	1	2	1	Q95KN9		0.0000	0	0.1731	2.70	3	1449.71006	-1.43	20.69	0
	High	GKAPAVFVK	1	2	1	Q95KN9		0.0000	0	0.5609	2.48	3	916.56131	-0.23	21.56	1

Spot 2

Accession	Description	Score	Coverage	# Proteins	# Unique Peptides	# Peptides	# PSMs	# AAs	MW [kDa]	calc. pI						
Q95KN9	Lipoprotein lipase OS=Macaca fascicularis PE=2 SV=1 - [Q95KN9_MACFA]	23.93	16.48	3	6	6	8	449	50.6	8.57						
	A9	Sequence	# PSMs	# Proteins	# Protein Groups	Protein Group Accessions	Modifications	ΔCn	q-Value	PEP	XCorr	Charge	MH+ [Da]	ΔM [ppm]	RT [min]	# Missed Cleavages
	High	ITGLDPAGPNFEYAEAPSR	2	3	1	Q95KN9		0.0000	0	0.000003542	4.35	2	2004.96721	0.48	28.79	0
	High	AQQHYPVSAGYTK	2	3	1	Q95KN9		0.0000	0	0.1364	3.89	2	1449.71294	0.56	20.67	0
	High	cNNLGYEINKVR	1	3	1	Q95KN9	C1(Carbamidomethyl)	0.0000	0	0.004358	3.43	2	1479.73821	0.60	24.46	1
	High	EPDSNVIVVDWLSR	1	3	1	Q95KN9		0.0000	0	0.2837	3.04	2	1628.83122	2.02	34.74	0
	High	GLcLScR	1	3	1	Q95KN9	C3(Carbamidomethyl); C6(Carbamidomethyl)	0.0000	0	0.2976	1.61	2	865.40166	-0.29	23.06	0
	High	GKAPAVFVK	1	2	1	Q95KN9		0.0000	0	0.06489	1.24	2	916.56114	-0.41	21.55	1
G7PUP9	Cytokeratin-10 (Fragment) OS=Macaca fascicularis GN=EGM_07881 PE=3 SV=1 - [G7PUP9_MACFA]	18.38	12.70	1	8	8	8	575	57.8	5.11						
G7PID3	Cytokeratin-2e OS=Macaca fascicularis GN=EGM_03270 PE=3 SV=1 - [G7PID3_MACFA]	13.39	7.02	1	3	4	4	641	65.5	8.02						
G7PIC8	Cytokeratin-6C OS=Macaca fascicularis GN=EGM_03263 PE=3 SV=1 - [G7PIC8_MACFA]	7.78	5.14	2	2	3	3	564	59.9	8.00						

Spot 3

Accession	Description	Score	Coverage	# Proteins	# Unique Peptides	# Peptides	# PSMs	# AAs	MW [kDa]	calc. pI						
Q95KN9	Lipoprotein lipase OS=Macaca fascicularis PE=2 SV=1 - [Q95KN9_MACFA]	43.12	25.17	3	12	12	16	449	50.6	8.57						
	A9	Sequence	# PSMs	# Proteins	# Protein Groups	Protein Group Accessions	Modifications	ΔCn	q-Value	PEP	XCorr	Charge	MH+ [Da]	ΔM [ppm]	RT [min]	# Missed Cleavages
	High	LSPDDADFVDVLHTFTR	2	3	1	Q95KN9		0.0000	0	0.0004259	4.82	2	1947.95122	3.31	35.52	0
	High	ITGLDPAGPNFEYAEAPSR	2	3	1	Q95KN9		0.0000	0	9.25674E-08	4.74	2	2004.96819	0.97	28.78	0
	High	AQQHYPVSAGYTK	2	3	1	Q95KN9		0.0000	0	0.004695	3.75	2	1449.71330	0.81	20.72	0
	High	SIHLFIDSLLNEENPSK	1	3	1	Q95KN9		0.0000	0	0.0002285	3.60	3	1956.00821	0.44	34.50	0
	High	cNNLGYEINKVR	2	3	1	Q95KN9	C1(Carbamidomethyl)	0.0000	0	0.001204	3.49	3	1479.73569	-1.10	24.50	1
	High	cNNLGYEINK	1	3	1	Q95KN9	C1(Carbamidomethyl)	0.0000	0	0.007676	2.89	2	1224.56755	-0.19	23.64	0
	High	ITGLDPAGPNFEYAEAPSRLSPDDADFVDVLHTF	1	3	1	Q95KN9		0.0000	0	0.03623	2.80	4	3933.90590	3.24	35.95	1
	High	NRcNNLGYEINK	1	3	1	Q95KN9	C3(Carbamidomethyl)	0.0000	0	0.00385	2.65	3	1494.70987	-1.31	22.02	1
	High	GLGDVDQLVK	1	3	1	Q95KN9		0.0000	0	0.0126	2.54	2	1043.57244	-0.74	27.64	0
	High	LVGQDVAR	1	3	1	Q95KN9		0.0000	0	0.008996	2.29	2	857.48357	-0.49	20.27	0
	High	KVIFcSR	1	2	1	Q95KN9	C5(Carbamidomethyl)	0.0000	0	0.1384	1.48	2	909.49657	-1.05	21.17	1
	High	LVAALYKR	1	3	1	Q95KN9		0.0000	0	0.1183	1.48	3	933.58792	-0.14	22.73	1
G7PUP9	Cytokeratin-10 (Fragment) OS=Macaca fascicularis GN=EGM_07881 PE=3 SV=1 - [G7PUP9_MACFA]	27.66	16.52	1	8	10	10	575	57.8	5.11						
G7PID3	Cytokeratin-2e OS=Macaca fascicularis GN=EGM_03270 PE=3 SV=1 - [G7PID3_MACFA]	21.38	11.54	1	6	8	9	641	65.5	8.02						
G7PUS5	Putative uncharacterized protein OS=Macaca fascicularis GN=EGM_07909 PE=3 SV=1 - [G7PUS5_MACF/	11.29	9.24	1	2	4	4	476	51.7	5.10						
G7PIC8	Cytokeratin-6C OS=Macaca fascicularis GN=EGM_03263 PE=3 SV=1 - [G7PIC8_MACFA]	7.75	7.09	2	2	4	4	564	59.9	8.00						
G7NV40	Alpha-amylase (Fragment) OS=Macaca fascicularis GN=EGM_00888 PE=3 SV=1 - [G7NV40_MACFA]	5.14	21.24	1	3	3	3	226	25.5	6.74						

Spot 4

Accession	Description	Score	Coverage	# Proteins	# Unique Peptides	# Peptides	# PSMs	# AAs	MW [kDa]	calc. pI						
Q95KN9	Lipoprotein lipase OS=Macaca fascicularis PE=2 SV=1 - [Q95KN9_MACFA]	56.23	42.54	3	16	16	20	449	50.6	8.57						
	A9	Sequence	# PSMs	# Proteins	# Protein Groups	Protein Group Accessions	Modifications	ΔCn	q-Value	PEP	XCorr	Charge	MH+ [Da]	ΔM [ppm]	RT [min]	# Missed Cleavages
	High	SIGIQKPVGHVDIYPNGGTFQPGcNIGEAIR	1	3	1	Q95KN9	C24(Carbamidomethyl)	0.0000	0	0.000002831	5.69	3	3294.67270	1.14	28.67	0
	High	SIHLFIDSLLNEENPSK	2	3	1	Q95KN9		0.0000	0	0.00007461	5.63	2	1956.00871	0.69	34.44	0
	High	ITGLDPAGPNFEYAEAPSR	2	3	1	Q95KN9		0.0000	0	0.0001491	4.77	2	2004.96831	1.03	28.80	0
	High	LSPDDADFVDVLHTFTR	2	3	1	Q95KN9		0.0000	0	0.0001349	4.71	2	1947.94744	1.37	35.40	0
	High	cNNLGYEINKVR	2	3	1	Q95KN9	C1(Carbamidomethyl)	0.0000	0	0.01819	4.03	3	1479.73633	-0.67	24.54	1
	High	EPDSNVIVVDWLSR	1	3	1	Q95KN9		0.0000	0	0.0446	3.38	2	1628.82805	0.07	34.75	0
	High	NRcNNLGYEINK	1	3	1	Q95KN9	C3(Carbamidomethyl)	0.0000	0	0.02733	3.07	2	1494.71172	-0.08	22.08	1
	High	SQMPYKVFHYQVK	1	3	1	Q95KN9		0.0000	0	0.0003778	2.98	3	1654.83982	-0.75	25.61	1
	High	GLGDVDQLVK	1	3	1	Q95KN9		0.0000	0	0.02865	2.84	2	1043.57170	-1.45	27.66	0
	High	AQQHYPVSAGYTK	1	3	1	Q95KN9		0.0000	0	0.005893	2.77	3	1449.71024	-1.31	20.69	0
	High	GLGDVDQLVKcSHER	1	3	1	Q95KN9	C11(Carbamidomethyl)	0.0000	0	7.705E-07	2.33	3	1712.83701	-0.89	25.69	1
	High	VIAERGLGDVDQLVK	1	3	1	Q95KN9		0.0000	0	0.08779	2.18	3	1611.90543	-0.69	27.73	1
	High	LVGQDVAR	1	3	1	Q95KN9		0.0000	0	0.1094	2.00	2	857.48332	-0.78	20.30	0
	High	GLcLScRK	1	3	1	Q95KN9	C3(Carbamidomethyl); C6(Carbamidomethyl)	0.0000	0	0.05916	1.60	3	993.49646	-0.42	20.72	1
	High	GKAPAVFVK	1	2	1	Q95KN9		0.0000	0	0.113	1.57	2	916.56090	-0.68	21.68	1
	High	LVAALYKR	1	3	1	Q95KN9		0.0000	0	0.004971	1.27	3	933.58802	-0.04	22.74	1
G7PUS5	Putative uncharacterized protein OS=Macaca fascicularis GN=EGM_07909 PE=3 SV=1 - [G7PUS5_MACF/	15.68	12.39	1	3	5	5	476	51.7	5.10						
G7PUP9	Cytokeratin-10 (Fragment) OS=Macaca fascicularis GN=EGM_07881 PE=3 SV=1 - [G7PUP9_MACFA]	15.38	10.78	1	4	6	6	575	57.8	5.11						
G7PID3	Cytokeratin-2e OS=Macaca fascicularis GN=EGM_03270 PE=3 SV=1 - [G7PID3_MACFA]	10.68	6.86	1	3	4	4	641	65.5	8.02						
G7NV40	Alpha-amylase (Fragment) OS=Macaca fascicularis GN=EGM_00888 PE=3 SV=1 - [G7NV40_MACFA]	7.85	22.12	1	3	3	3	226	25.5	6.74						
G7PIE0	Putative uncharacterized protein (Fragment) OS=Macaca fascicularis GN=EGM_03280 PE=3 SV=1 - [G7I	5.15	6.41	1	2	3	3	468	51.8	5.40						

Spot 5

Accession	Description	Score	Coverage	# Proteins	# Unique Peptides	# Peptides	# PSMs	# AAs	MW [kDa]	calc. pI
G7PID3	Cytokeratin-2e OS=Macaca fascicularis GN=EGM_03270 PE=3 SV=1 - [G7PID3_MACFA]	26.81	14.35	1	10	10	10	641	65.5	8.02
G7PCN4	Lipoprotein lipase OS=Macaca fascicularis GN=EGM_17127 PE=3 SV=1 - [G7PCN4_MACFA]	26.10	15.65	3	7	7	9	441	49.4	7.55

A9	Sequence	# PSMs	# Proteins	# Protein Groups	Protein Group Accessions	Modifications	ΔCn	q-Value	PEP	XCorr	Charge	MH+ [Da]	ΔM [ppm]	RT [min]	# Missed Cleavages
High	ITGLDPAGPNFEYAEAPSR	1	3	1	G7PCN4		0.0000	0	0.2491	4.49	2	2004.96929	1.52	28.88	0
High	cNNLGYEINKVR	2	3	1	G7PCN4	C1(Carbamidomethyl)	0.0000	0	0.1418	3.83	3	1479.73624	-0.73	24.53	1
High	AQQHYPVSAGYTK	2	3	1	G7PCN4		0.0000	0	0.3906	3.60	2	1449.71245	0.22	20.81	0
High	EPDSNVIVVDWLSR	1	3	1	G7PCN4		0.0000	0	0.3315	3.43	2	1628.82891	0.59	34.98	0
High	cNNLGYEINK	1	3	1	G7PCN4	C1(Carbamidomethyl)	0.0000	0	0.2461	2.61	2	1224.56755	-0.19	23.66	0
High	NRcNNLGYEINK	1	3	1	G7PCN4	C3(Carbamidomethyl)	0.0000	0	0.3707	2.03	3	1494.70960	-1.50	22.06	1
High	RDFIDIESK	1	3	1	G7PCN4		0.0000	0	0.4278	1.45	2	1122.57793	-0.97	25.93	1

G7PUP9	Cytokeratin-10 (Fragment) OS=Macaca fascicularis GN=EGM_07881 PE=3 SV=1 - [G7PUP9_MACFA]	25.10	16.35	1	8	9	10	575	57.8	5.11
G7PUS5	Putative uncharacterized protein OS=Macaca fascicularis GN=EGM_07909 PE=3 SV=1 - [G7PUS5_MACF	9.70	8.19	1	2	3	3	476	51.7	5.10

Spot 6

Accession	Description	Score	Coverage	# Proteins	# Unique Peptides	# Peptides	# PSMs	# AAs	MW [kDa]	calc. pI
G7PID3	Cytokeratin-2e OS=Macaca fascicularis GN=EGM_03270 PE=3 SV=1 - [G7PID3_MACFA]	41.80	20.44	1	15	15	18	641	65.5	8.02
G7PUP9	Cytokeratin-10 (Fragment) OS=Macaca fascicularis GN=EGM_07881 PE=3 SV=1 - [G7PUP9_MACFA]	40.59	22.78	1	12	14	17	575	57.8	5.11
Q95KN9	Lipoprotein lipase OS=Macaca fascicularis PE=2 SV=1 - [Q95KN9_MACFA]	35.04	24.72	3	12	12	14	449	50.6	8.57

A9	Sequence	# PSMs	# Proteins	# Protein Groups	Protein Group Accessions	Modifications	ΔCn	q-Value	PEP	XCorr	Charge	MH+ [Da]	ΔM [ppm]	RT [min]	# Missed Cleavages
High	ITGLDPAGPNFEYAEAPSR	1	3	1	Q95KN9		0.0000	0	0.00002557	4.64	2	2004.96819	0.97	28.94	0
High	AQQHYPVSAGYTK	2	3	1	Q95KN9		0.0000	0	0.151	4.27	2	1449.71269	0.39	20.84	0
High	cNNLGYEINKVR	1	3	1	Q95KN9	C1(Carbamidomethyl)	0.0000	0	0.1179	3.83	3	1479.73670	-0.42	24.68	1
High	EPDSNVIVVDWLSR	1	3	1	Q95KN9		0.0000	0	0.0001478	3.64	2	1628.83013	1.34	34.98	0
High	cNNLGYEINK	1	3	1	Q95KN9	C1(Carbamidomethyl)	0.0000	0	0.01572	2.87	2	1224.56792	0.11	23.73	0
High	GLGDVDQLVK	1	3	1	Q95KN9		0.0000	0	0.151	2.60	2	1043.57292	-0.28	27.77	0
High	NRcNNLGYEINK	2	3	1	Q95KN9	C3(Carbamidomethyl)	0.0000	0	0.0006218	2.49	2	1494.71306	0.82	22.18	1
High	LVGQDVAR	1	3	1	Q95KN9		0.0000	0	0.006309	2.30	2	857.48326	-0.85	20.34	0
High	DFIDIESK	1	3	1	Q95KN9		0.0000	0	0.03216	1.92	2	966.47722	-0.70	28.00	0
High	LVAALYKR	1	3	1	Q95KN9		0.0000	0	0.00392	1.80	3	933.58774	-0.33	22.90	1
High	GLcLScRK	1	3	1	Q95KN9	C3(Carbamidomethyl); C6(Carbamidomethyl)	0.0000	0	0.07978	1.77	2	993.49626	-0.62	21.01	1
High	GKAPAVFVK	1	2	1	Q95KN9		0.0000	0	0.112	1.51	2	916.56096	-0.61	21.76	1

G7PUS5	Putative uncharacterized protein OS=Macaca fascicularis GN=EGM_07909 PE=3 SV=1 - [G7PUS5_MACF	15.14	9.24	1	2	4	6	476	51.7	5.10
G8F3F7	Putative uncharacterized protein OS=Macaca fascicularis GN=EGM_19949 PE=4 SV=1 - [G8F3F7_MACF	5.63	3.28	1	2	2	2	579	64.3	8.41
I7G2V3	Macaca fascicularis brain cDNA clone: QmoA-12188, similar to human similar to FLJ27099 protein (LOC3	5.46	6.00	2	2	2	2	433	47.2	8.59
G7P6F7	Putative uncharacterized protein OS=Macaca fascicularis GN=EGM_14761 PE=4 SV=1 - [G7P6F7_MACF	1.99	5.70	1	2	2	2	491	55.7	8.70

Spot 7

Accession	Description	Score	Coverage	# Proteins	# Unique Peptides	# Peptides	# PSMs	# AAs	MW [kDa]	calc. pI
G7PID3	Cytokeratin-2e OS=Macaca fascicularis GN=EGM_03270 PE=3 SV=1 - [G7PID3_MACFA]	42.80	18.56	1	15	15	19	641	65.5	8.02
G7PUP9	Cytokeratin-10 (Fragment) OS=Macaca fascicularis GN=EGM_07881 PE=3 SV=1 - [G7PUP9_MACFA]	31.69	17.91	1	10	12	13	575	57.8	5.11
Q95KN9	Lipoprotein lipase OS=Macaca fascicularis PE=2 SV=1 - [Q95KN9_MACFA]	29.58	20.71	3	9	9	11	449	50.6	8.57

A9	Sequence	# PSMs	# Proteins	# Protein Groups	Protein Group Accessions	Modifications	ΔCn	q-Value	PEP	XCorr	Charge	MH+ [Da]	ΔM [ppm]	RT [min]	# Missed Cleavages
High	ITGLDPAGPNFEYAEAPSR	1	3	1	Q95KN9		0.0000	0	1.412E-08	4.64	2	2004.96807	0.91	28.98	0
High	AQQHYPVSAGYTK	2	3	1	Q95KN9		0.0000	0	6.625E-07	4.18	2	1449.71318	0.73	20.94	0
High	cNNLGYEINKVR	2	3	1	Q95KN9	C1(Carbamidomethyl)	0.0000	0	0.003976	3.76	3	1479.73670	-0.42	24.72	1
High	EPDSNVIVVDWLSR	1	3	1	Q95KN9		0.0000	0	6.562E-08	3.48	2	1628.82976	1.12	34.95	0
High	GLGDVDQLVK	1	3	1	Q95KN9		0.0000	0	0.0003426	2.39	2	1043.57207	-1.09	27.83	0
High	LVGQDVAR	1	3	1	Q95KN9		0.0000	0	0.05006	2.33	2	857.48369	-0.35	20.41	0
High	cNNLGYEINK	1	3	1	Q95KN9	C1(Carbamidomethyl)	0.0000	0	0.00001689	2.30	2	1224.56755	-0.19	23.83	0
High	GKAPAVFVK	1	2	1	Q95KN9		0.0000	0	0.2106	1.43	3	916.56104	-0.53	21.75	1
High	LVAALYKR	1	3	1	Q95KN9		0.0000	0	0.02092	1.33	3	933.58783	-0.23	22.92	1

G7PUS5	Putative uncharacterized protein OS=Macaca fascicularis GN=EGM_07909 PE=3 SV=1 - [G7PUS5_MACF	11.83	9.66	1	2	4	4	476	51.7	5.10

Spot 8

Accession	Description	Score	Coverage	# Proteins	# Unique Peptides	# Peptides	# PSMs	# AAs	MW [kDa]	calc. pI
G7P6F7	Putative uncharacterized protein OS=Macaca fascicularis GN=EGM_14761 PE=4 SV=1 - [G7P6F7_MACF	34.59	24.24	1	13	13	15	491	55.7	8.70
G7PUP9	Cytokeratin-10 (Fragment) OS=Macaca fascicularis GN=EGM_07881 PE=3 SV=1 - [G7PUP9_MACFA]	30.29	17.22	1	8	11	12	575	57.8	5.11
G7PID3	Cytokeratin-2e OS=Macaca fascicularis GN=EGM_03270 PE=3 SV=1 - [G7PID3_MACFA]	29.32	14.98	1	10	10	11	641	65.5	8.02
G7PUS5	Putative uncharacterized protein OS=Macaca fascicularis GN=EGM_07909 PE=3 SV=1 - [G7PUS5_MACF	16.50	13.45	1	3	6	6	476	51.7	5.10
G7PCN4	Lipoprotein lipase OS=Macaca fascicularis GN=EGM_17127 PE=3 SV=1 - [G7PCN4_MACFA]	12.48	11.34	3	4	4	4	441	49.4	7.55

A9	Sequence	# PSMs	# Proteins	# Protein Groups	Protein Group Accessions	Modifications	ΔCn	q-Value	PEP	XCorr	Charge	MH+ [Da]	ΔM [ppm]	RT [min]	# Missed Cleavages
High	ITGLDPAGPNFEYAEAPSR	1	3	1	G7PCN4		0.0000	0	5.349E-16	4.57	2	2004.96733	0.54	29.00	0
High	LVGQDVAR	1	3	1	G7PCN4		0.0000	0	0.0001778	2.68	2	857.48338	-0.71	20.39	0
High	GLGDVDQLVK	1	3	1	G7PCN4		0.0000	0	0.000003082	2.67	2	1043.57231	-0.86	27.83	0
High	AQQHYPVSAGYTK	1	3	1	G7PCN4		0.0000	0	4.679E-14	2.56	3	1449.70987	-1.56	20.84	0

I7G2V3	Macaca fascicularis brain cDNA clone: QmoA-12188, similar to human similar to FLJ27099 protein (LOC3	5.52	6.00	2	2	2	2	433	47.2	8.59

Spot 9

Accession	Description	Score	Coverage	# Proteins	# Unique Peptides	# Peptides	# PSMs	# AAs	MW [kDa]	calc. pI						
G7PID3	Cytokeratin-2e OS=Macaca fascicularis GN=EGM_03270 PE=3 SV=1 - [G7PID3_MACFA]	31.42	14.66	1	9	9	10	641	65.5	8.02						
G7P6F7	Putative uncharacterized protein OS=Macaca fascicularis GN=EGM_14761 PE=4 SV=1 - [G7P6F7_MACF/	21.57	15.68	1	8	8	9	491	55.7	8.70						
G7PUP9	Cytokeratin-10 (Fragment) OS=Macaca fascicularis GN=EGM_07881 PE=3 SV=1 - [G7PUP9_MACFA]	20.03	13.57	1	6	7	8	575	57.8	5.11						
G7PCN4	Lipoprotein lipase OS=Macaca fascicularis GN=EGM_17127 PE=3 SV=1 - [G7PCN4_MACFA]	15.81	12.47	3	5	5	5	441	49.4	7.55						
	A9	Sequence	# PSMs	# Proteins	# Protein Groups	Protein Group Accessions	Modifications	ΔCn	q-Value	PEP	XCorr	Charge	MH+ [Da]	ΔM [ppm]	RT [min]	# Missed Cleavages
	High	ITGLDPAGPNFEYAEAPSR	1	3	1	G7PCN4		0.0000	0	0.0008145	4.24	2	2004.96831	1.03	29.11	0
	High	cNNLGYEINK	1	3	1	G7PCN4	C1(Carbamidomethyl)	0.0000	0	0.09493	3.19	2	1224.56792	0.11	23.93	0
	High	EPDSNVIVVDWLSR	1	3	1	G7PCN4		0.0000	0	0.003852	2.96	2	1628.82854	0.37	35.14	0
	High	cNNLGYEINKVR	1	3	1	G7PCN4	C1(Carbamidomethyl)	0.0000	0	0.00244	2.90	2	1479.73821	0.60	24.84	1
	High	GLGDVDQLVK	1	3	1	G7PCN4		0.0000	0	0.06637	2.53	2	1043.57280	-0.39	27.89	0
G7PUS5	Putative uncharacterized protein OS=Macaca fascicularis GN=EGM_07909 PE=3 SV=1 - [G7PUS5_MACF/	14.33	10.50	1	3	4	4	476	51.7	5.10						
