# Supplementary material for: The response to fasting and refeeding reveals functional regulation of lipoprotein lipase proteoforms
Source: Front Physiol. 2023 Oct 16;14:1271149. doi: 10.3389/fphys.2023.1271149 (PMC10617031; doi:10.3389/fphys.2023.1271149)

frontiers

Supplementary Material

The response to fasting and refeeding reveals functional regulation of lipoprotein lipase proteoforms

Pere Carulla[1], Míriam Badia-Villanueva[1], Sergi Civit[2], Montserrat Carrascal[3], Joaquín Abián[3], David Ricart-Jané[1], Miquel Llobera[1], Albert Casanovas[1,†,*], M. Dolores López-Tejero[1,†,*]

[1]Departament de Bioquímica i Biomedicina Molecular, Facultat de Biologia, Universitat de Barcelona (UB), Diagonal 643, 08028 Barcelona, Spain

[2]Departament de Genètica, Microbiologia i Estadística, Facultat de Biologia, Universitat de Barcelona (UB), Diagonal 643, 08028 Barcelona, Spain

[3]Biological and Environmental Proteomics, Institute of Biomedical Research of Barcelona, Spanish National Research Council, Institut d'Investigacions Biomèdiques August Pi i Sunyer (IIBB-CSIC/ IDIBAPS), Barcelona, Spain

†These authors contributed equally to this work and share last authorship.

*Correspondence:

M. Dolores López-Tejero
E-mail: dolopez@ub.edu

Albert Casanovas
E-mail: acasanto@gmail.com

Contents:

Supplementary Figures

Figure S1. Partial purification of LPL from rat tissues.

Figure S2. Overlap of LPL proteoforms of rat WAT from different physiological conditions.

Figure S3. LPL proteoforms from cynomolgus monkey WAT.

Supplementary Tables

Table S1. Identification of proteins from Figure S3 by LC-MS/MS.

(Supplementary Table S1 is supplied in a separate PDF file)

Supplementary Figures

Figure S1. Partial purification of LPL from rat tissues. (A) LPL was partially purified from tissue homogenates using heparin-Sepharose affinity chromatography. Fractions were collected after stepwise increase in NaCl concentration as indicated at the top. Total protein (white circles) and LPL activity (black circles) were quantified in fractions. Partial purification of LPL from rat heart is shown as a representative example. (B) LPL-containing fractions eluted with 1.5 M NaCl were pooled and analyzed by 2DE and silver staining (left) or 2DE Western blot against LPL (right) in heart, muscle, WAT and BAT tissues. The arrow indicates the apparent MW of LPL (56 kDa).

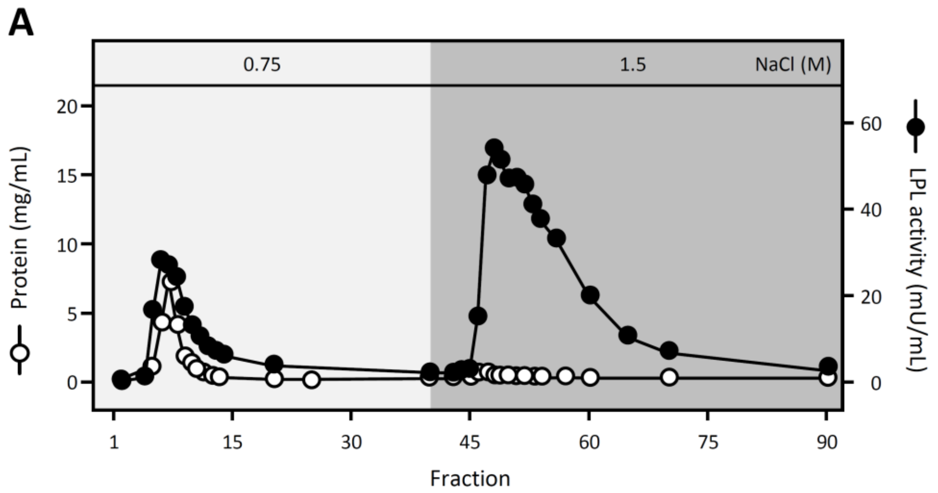

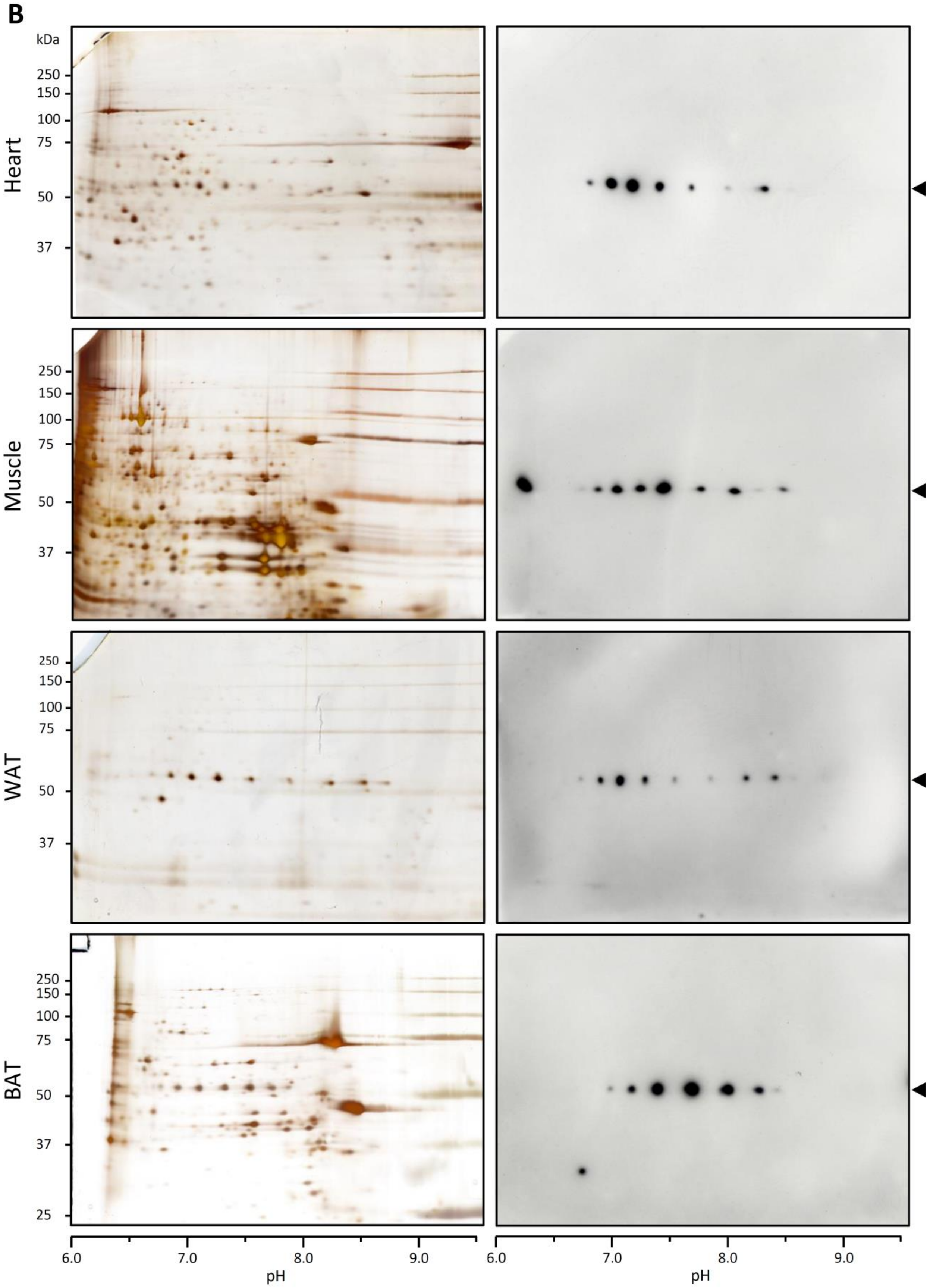

B
kDa
250
150
100
75
50
37
25
Heart
Muscle
WAT
BAT
6.0
7.0
8.0
9.0
pH

Figure S2. Overlap of LPL proteoforms of rat WAT from different physiological conditions. Partially purified LPL from rat WAT of control, fasting and refeeding groups was analyzed by difference gel electrophoresis (DIGE) to confirm that the pI of LPL proteoforms does not differ between conditions. In DIGE, each sample is labeled with a specific fluorescent dye and can be viewed separately or superimposed for comparison between samples. (A) Diagram for color interpretation. (B) DIGE of individual (top) or superimposed (bottom) samples. The arrow indicates the apparent MW of LPL (56 kDa).

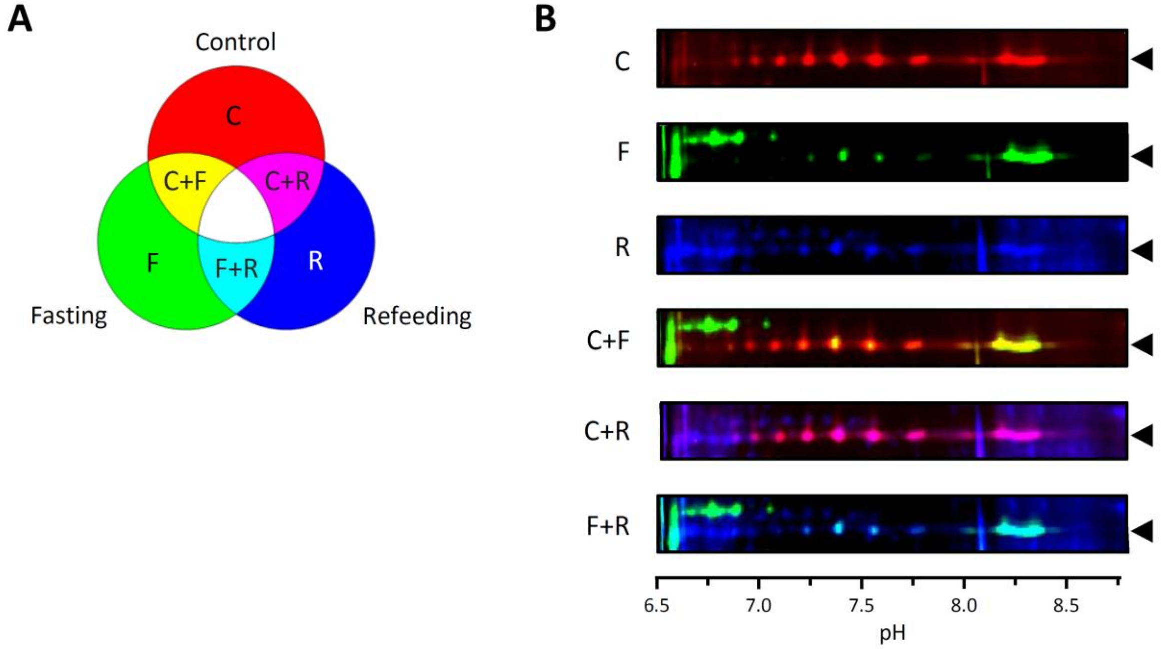

Figure S3. LPL proteoforms from cynomolgus monkey WAT. (A) LPL was partially purified from visceral WAT of cynomolgus monkey using heparin-Sepharose affinity chromatography and (B) analyzed by 2DE and silver staining (left) or 2DE Western blot against LPL (right). Spots excised from the silver-stained gel and identified as LPL by LC-MS/MS are numbered (see Table S1). The arrow indicates the apparent MW of LPL (56 kDa).

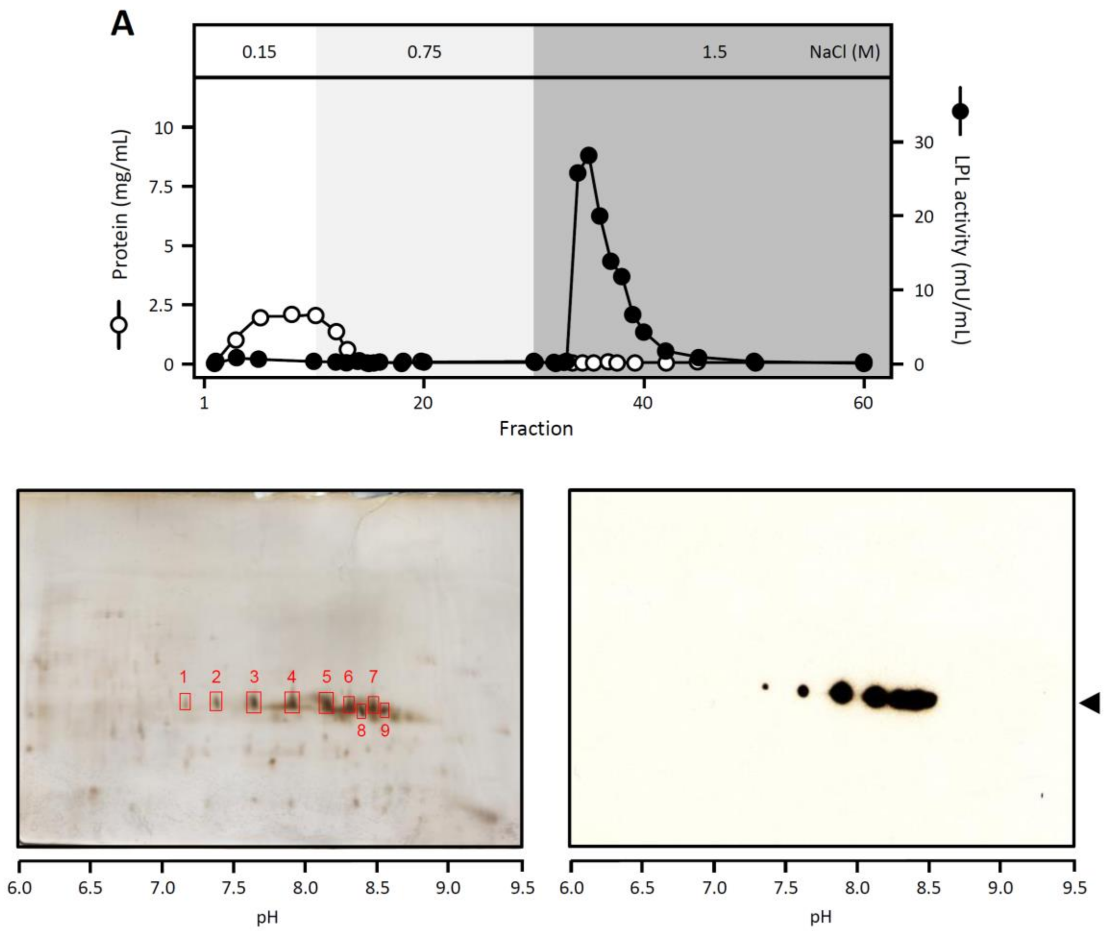

Supplement: Supplementary file 2 [file DataSheet1.PDF]
